# PIM1 kinase facilitates Zika virus replication by suppressing host cells’ natural immunity

**DOI:** 10.1038/s41392-021-00539-x

**Published:** 2021-06-02

**Authors:** Fanghang Zhou, Qianya Wan, Ying Chen, Sheng Chen, Ming-liang He

**Affiliations:** 1 Department of Biomedical Sciences, City University of Hong Kong, Kowloon, Hong Kong SAR China; 2 Department of Biomedical Sciences, College of Veterinary Medicine, Cornell University, Ithaca, NY USA; 3 CityU Shenzhen Research Institute, Nanshan, Shenzhen, China

**Keywords:** Innate immunity, Infection

**Dear Editors,**

Oncoprotein PIM1 kinase participates in many important biological processes, such as cell proliferation, apoptosis, carcinogenesis and tumorigenesis, by phosphorylating cellular substrates. More recently, several groups discovered that PIM1 affects (+) ssRNA virus transcription and modulates virus infection, such as human rhinovirus (HRV)-16^1^ and hepatitis C virus (HCV).^2^ We recently reported that PIM1 enhances EV-A71 IRES activity by regulating AUF1 translocation.^3^ However, its function in ZIKV infection has not been explored. Interestingly, after we revisited unbiased RNA-sequencing data obtained from ZIKV-infected Vero cells^4^ and further analyzed the genes with significant changes; the results from the KEGG pathway showed that ZIKV infection affects many pathways, including pathways in cancer, virus infection, viral carcinogenesis, spliceosome, and the cell cycle (Supplementary Fig. 1a). The upregulation of PIM1 expression attracted our attention (Fig. 1a) because it had a pattern similar to that of infection with the (+)ssRNA virus EV-A71 and exhibited important roles in EV-A71 reproduction in another study.^3^

**Fig. 1:**
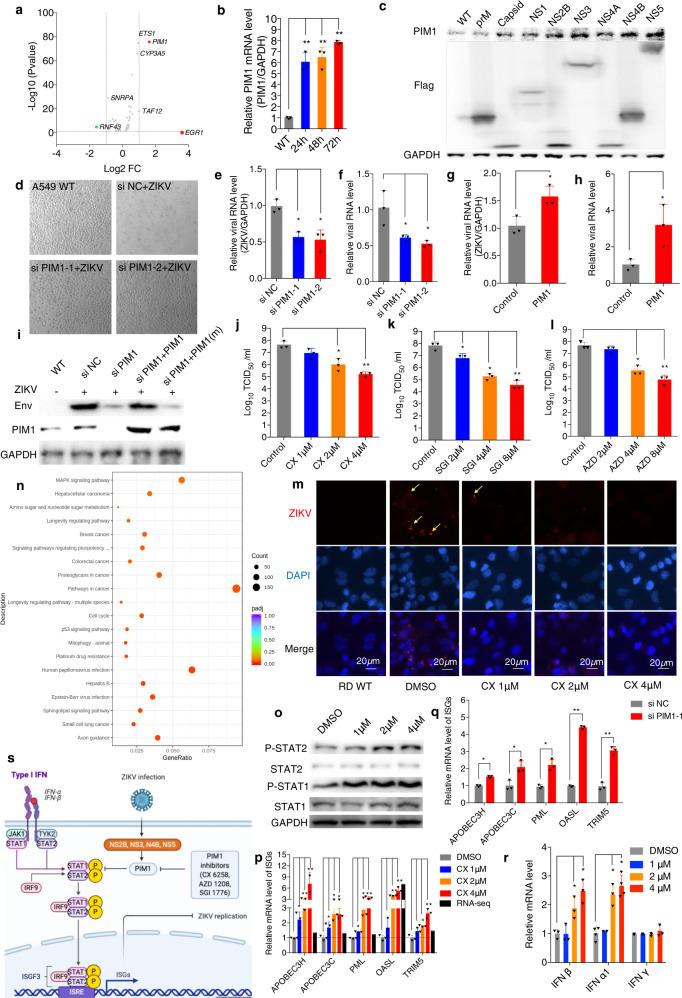
**a** The volcano plot of differentially expressed genes after ZIKV infection. **b** RD cells were infected with ZIKV at the indicated MOI of 1 and incubated for the indicated time points, and cellular mRNA was extracted at different time points h.p.i.; the mRNA level of PIM1was determined by RT-qPCR. GAPDH was used as the internal control. **c** Different ZIKV proteins with flag tag (prM, capsid, NS1, NS2B, NS3, NS4A, NS4B, and NS5) were ectopically expressed in HEK293T cells for 48 h. The protein levels of PIM1 and ZIKV proteins were determined by WB. **d** The cytopathic effects of A549 cells with and without PIM1 knockdown after ZIKV infection at MOI = 1 at 48 h. **e**, **f** PIM1 was knocked down in A549 cells, which were infected with ZIKV at an MOI of 1 for 48 h. The levels of intracellular ZIKV RNA (**e**) and the extracellular virion RNA (**f**) were determined by RT-qPCR assay. **g**, **h** PIM1 was ectopically expressed in A549 cells, which were infected with ZIKV at an MOI of 1 for 48 h. The levels of intracellular ZIKV RNA (g) and the extracellular virion RNA (h) were determined by RT-qPCR assay. **i** siRNA targeting the PIM1 3’-UTR at 40 nM was cotransfected with the PIM1 or PIM1 mutant (K67M) expression plasmid in RD cells, which were infected 48 h later with ZIKV at an MOI of 1 and incubated for additional 48 h. ZIKV envelope protein expression level was determined. RD cells were also treated with CX-6258 **j**, SGI-1776 **k** and AZD-1208 **l** at the indicated concentrations for 2 h and then infected with ZIKV at an MOI of 0.1 and incubated for 72 h. The viral titer was measured by TCID_50_ assay. **m** RD cells were treated with CX-6258 at the indicated concentrations for 2 h and then infected with ZIKV at an MOI of 1 and incubated for 24 h. The viral protein envelope was observed by fluorescence microscopy. Red represents the ZIKV envelope protein, and blue is DAPI. **n** KEGG pathway enrichment analysis of the RNA-sequencing results. **o**, **p**, **r** HEK293T cells were treated for 24 h with CX-6258 at the indicated concentrations. The relative phosphorylation of STAT1 and STAT2 was determined by WB (**o**); the relative mRNA levels of ISGs (APOBEC3H, APOBEC3C, PML, OASL and TRIM5) (**q**), and IFN β, IFN α1 and IFN γ (**r**) were determined by RT-qPCR. **s** The mechanism by which PIM1 mediates ZIKV replication (Created with BioRender.com). Data are presented as the means ± SD (*n* = 3). Student’s *t*-test, *p < 0.05, compared with the mock group; **p < 0.01, compared with the mock group

To confirm our findings, we examined the mRNA levels of PIM1 in RD cells 48 h postinfection (h.p.i.) with ZIKV at a multiplicity of infection (MOI) of 1. The results showed that the mRNA level of PIM1 was upregulated at least 6-fold between 24 h and 72 h after ZIKV infection (Fig. 1b). We also observed that PIM1 protein levels were significantly upregulated in both RD and A549 cells 48 h after ZIKV infection (Supplementary Fig. 1b, c). We ectopically expressed ZIKV structure proteins (prM, capsid) and nostructure proteins in HEK293T cells for 48 h. Almost all the tested ZIKV proteins (except prM) induced PIM1 expression; in particular, NS3, NS4B, and NS5 strongly stimulated PIM1 expression (Fig. 1c).

To reveal the role of PIM1 in ZIKV infection, we knocked down PIM1 by using two different siRNAs (Supplementary Fig. 2a, b). Forty-eight hours after siRNA transfection, we infected the cells with ZIKV at MOI of 1. PIM1 knockdown protected A549 cells from the cytopathic effects (CPE) induced by ZIKV infection (Fig. 1d). The expression level of a viral protein (envelope) was significantly decreased in the PIM1-depleted RD and A549 cells (Supplementary Fig. 2c, d). The ectopic expression of PIM1 strongly increased envelope protein expression in both the RD and A549 cells (Supplementary Fig. 2e, f). Intriguingly, both the intracellular and extracellular viral RNA levels were decreased in the PIM1-depleted RD and A549 cells 24 h.p.i. (Fig. 1e, f, Supplementary Fig. 2g, h). Obviously, the levels of both the intracellular viral RNA and extracellular virion RNA were significantly increased in the RD and A549 cells with ectopically expressed PIM1 24 h.p.i. (Fig. 1g, h, Supplementary Fig. 2i, j). Examining the immunofluorescence assay results, we observed loci with strong viral replication, whereas no obvious ZIKV replication loci were detected in the PIM1-depleted RD cells 24 h.p.i. (Supplementary Fig. 2k). To determine whether PIM1 kinase activity is essential for facilitating ZIKV replication, we first knocked down the endogenous PIM1 with a siRNA targeting the 3’-UTR and then ectopically expressed PIM1 or a kinase-inactivated PIM1 mutant (K67M) in RD cells for 48 h and then infected them with ZIKV at an MOI of 1 and measured the levels after 24 h. Our results showed that the reduced envelope protein level caused by PIM1 depletion was completely restored by the ectopic PIM1 expression, but no restoration was observed in the cells with ectopic expression of K67M mutant (Fig. 1i). In addition, the expression levels of NS5 and NS1 were also rescued by ectopically expressing PIM1 after ZIKV infection in the PIM1-depleted RD cells (Supplementary Fig. 2l).

The effects of PIM1 inhibitors (CX-6258, SGI-1776 and AZD-1208) were tested on ZIKV infection in both RD and A549 cells. We pretreated RD and A549 cells for 2 h with each inhibitor at different concentrations and then infected them with ZIKV at MOI of 1. Twenty-four hours later, we found that the envelope protein level was remarkably repressed by PIM1 inhibitors in a dose-dependent manner (Supplementary Fig. 3a–f). The intracellular replicated viral RNA level was also markedly decreased, by over 90%, in the cells treated with PIM1 inhibitors at a concentration of 8 μM (Supplementary Fig. 3g–i). More importantly, the viral titer was decreased more than 100- to 1000-fold by the PIM1 inhibitors (Fig. 1j-l). In addition, we directly observed the potent inhibition of PIM1-induced ZIKV replication by CX-6258 under a fluorescence microscope 24 h.p.i. (Fig. 1m).

To further address the mechanism by which PIM1 may be involved in promoting ZIKV replication, we treated HEK293T and A549 cells with CX-6258 at 4 μM for 24 h and took advantage of RNA-sequencing technology for transcriptome assays. Our results showed that the expression of many genes had significantly changed (Supplementary Fig. 4a and Supplementary Table). KEGG pathway assays showed that PIM1 is involved in many pathways mostly involved in cancer development, such as MARK signaling and p53 signaling (Fig. 1n). PIM1 was also found to be involved in many virus infection pathways, such as hepatitis B virus, Epstein-Barr virus and human papilloma virus infection (Fig. 1n). More interestingly, inhibition of PIM1 kinase activity upregulated important downstream genes associated with antiviral responses that play crucial roles in the cellular type I IFN signaling pathway for antiviral activity (Supplementary Table). APOBEC3H, APOBEC3C, PML, OASL and TRIM5 were found upregulated in both HEK293T and A549 cells. These interferon-stimulated genes (ISGs) were upregulated by CX-6258 in a dose-dependent manner in both HEK293T and A549 cells (Fig. 1p and Supplementary Fig. 4b). Similar results were also obtained by silencing PIM1 with siRNA in both HEK293T and A549 cells (Fig. 1q and Supplementary Fig. 4c).

The p-STAT1/p-STAT2 complex must undergo nuclear translocation to transactivate ISGs involved in antiviral activity. We then tested whether PIM1 affected the type I IFN signaling pathway by regulating the phosphorylation of STAT1 or STAT2. Our results showed that upon silencing of PIM1 phosphorylation of both STAT1 and STAT2 was upregulated in the HEK293T cells (Supplementary Fig. 4d), and similar results were also obtained after CX-6285 treatment (Fig. 1o). However, ectopic expression of PIM1 significantly decreased the phosphorylation levels of both STAT1 and STAT2 in HEK293T cells (Supplementary Fig. 4e). We also confirmed that the p-STAT2 protein level was increased and accumulated from cytoplasm to the nucleus after silencing PIM1 (Supplementary Fig. 4f). In addition, the mRNA levels of both IFNα and IFNβ were significantly increased by silencing PIM1, but not affecting IFN γ expression level (Supplementary Fig. 4g). In CX-6258-treated cells, the levels of both IFNα and IFNβ were also increased at a dose-dependent manner, but not affecting IFN γ expression level (Fig. 1r).

In conclusion, our study demonstrated that PIM1 is a negative regulator of type I IFN signaling during ZIKV infection (Fig. 1s). PIM1 inhibitors (SGI-1776, AZD-1208 and CX-6258) potently inhibit ZIKV reproduction, displaying great potential for use in anti-ZIKV therapeutics. Our findings may present a common mechanism for viruses to escape the host cells’ natural immunity, and target PIM1 kinase signaling would be effective for combating a batch of virus infections.

## Supplementary information

Supplementary tables

Supplementary materials

## Data Availability

The data sets used and/or analyzed during the current study are available from the corresponding author on reasonable request.

## References

[1] de Vries M (2015). Inhibition of Pim1 kinase reduces viral replication in primary bronchial epithelial cells. Eur. Respir. J..

[2] Park C (2015). Pim kinase interacts with nonstructural 5A protein and regulates hepatitis C virus entry. J. Virol..

[3] Zhou F (2019). Pim1 impacts enterovirus A71 replication and represents a potential target in antiviral therapy. iScience.

[4] Arunachalam Ramaiah, D. C., Gangalapudi, V., Padhye, M. S., Tang, J. & Arumugaswami, V. Dysregulation of long non-coding RNA (lncRNA) genes and predicted lncRNA-protein interactions during Zika virus infection. Preprint at https://www.biorxiv.org/content/10.1101/061788v1.full (2016).

