# Supplementary material for: PIM1 kinase facilitates Zika virus replication by suppressing host cells’ natural immunity
Source: Signal Transduct Target Ther. 2021 Jun 2;6:207. doi: 10.1038/s41392-021-00539-x (PMC8169747; doi:10.1038/s41392-021-00539-x)
Supplement: Supplementary file 2 — Supplementary materials [file 41392_2021_539_MOESM2_ESM.docx]

Supplementary Information for

**PIM1 Kinase Facilitates Zika Virus Evasion by Suppressing**

**Host Cells’ Natural Immunity**

Fanghang Zhou^1^, Qianya Wan^1,2^, Ying Chen^1^, Sheng Chen^1^; Ming-liang He^1, 3*^

^1^ Department of Biomedical Sciences, City University of Hong Kong, Kowloon, Hong Kong SAR, China.

^2^ Department of Biomedical Sciences, College of Veterinary Medicine, Cornell University, Ithaca, NY 14853, USA

^3^ CityU Shenzhen Research Institute, Nanshan, Shenzhen, China

*Correspondence Contact Information

Address: 1A-202, 2/F, Block 1, To Yuen Building

Phone: +852 3442-4492

Fax: +852 3442-0549

**Email:** mlhe7788@gmail.com

**This file includes:**

Materials and Methods;

Figure. s1-s4

**Methods and Materials**

**1. Cells and virus**

Cell lines of Rhabdomyosarcoma cells (RD), A549, Vero and HEK293T were purchased from ATCC^®^ (USA) and maintained in Dulbecco’s modified Eagle’s medium (DMEM) containing 10% fetal bovine serum (FBS) with 100 U/ml penicillin and 100 μg/ml streptomycin. Zika virus strain PRVABC-59 was purchased from ATCC^®^.

**2. PIM1 inhibitors**

CX-6258 ((3E)-5-chloro-3-[[5-[3-[(hexahydro-4-methyl-1H-1,4-diazepin-1-yl) carbonyl]phenyl]-2-furanyl]methylene]-1,3-dihydro-2H-indol-2-one), SGI-1776 (N-[(1-methyl-4-piperidinyl)methyl]-3-[3-(trifluoromethoxy)phenyl]-imidazo[1,2-b]pyridazin-6-amine) and AZD-1208 ((5Z)-[[2-[(3R)-3-amino-1-piperidinyl][1,1'-biphenyl]-3-yl]methylene]-2,4-thiazolidinedione) were purchased from MedChemExpress (USA).

**3. Real-Time Polymerase Chain Reaction**

The real-time PCR was carried out in the Applied Biosystems QuantStudio™ 3 Real-Time PCR Systems (Thermo Fisher Scientific, USA) with Power SYBR Green Master Mix (Applied Biosystems, USA), using the following program: 50 °C for 2 min, 95°C for 10 min followed by 45 cycles of 95°C for 15s and 60°C for 1 min. Sets of primers for these genes are available upon request. All samples were run in triplicate and the experiment was repeated at least three times. The messenger RNA (mRNA) levels of each target genes were normalized to the mRNA copies of GAPDH in the same sample and results were expressed as a percentage of the negative control (set as 1).

**4. RNA interference**

RNA interference was carried out using siRNAs purchased from Genepharma (ShangHai, China). siRNAs were used to inhibit endogenous protein expression. The specific siRNAs are listed below: siPim1-1 (sense): AACCUUCGAAGAAAUCCAGAACCAU; siPim1-2 (sense): GUAUGAUAUGGUGUGUGGAGAUAUUC, siRNA was designed by targeting Pim1 3’-UTR (sense: 5′-ACAUUUACAACUCAUUCCA-3′) (Park et al., 2015). Scramble siRNA was used as the control. Transfection of siRNA was performed according to the manufacturer’s instructions. In brief, cells at 50% confluence were transfected with 40 nM siRNA using the HiPerFect Transfection Reagent (QIAGEN, Germany) according to the manufacture instruction.

**5. Construction of Plasmids**

Plasmids contain the coding sequence of ZIKV viral proteins (prM, Capsid, NS1, NS2B, NS3, NS4A, NS4B, NS5) were purchased from Addgene (USA). Human PIM1 (Accession NM_001243186) was amplified using Platinums Taq DNA Polymerase (Invitrogen, USA). The PCR products were inserted into the MCS of pcDNA4/HisMax B (Invitrogen, USA) vector between BamH I and Xba I sites. The kinase inactive PIM1 mutant was constructed as described in the previous reporting (Chen et al., 2016). The dominant-negative K67M mutant of PIM1was generated by PCR methods using pfu DNA Polymerase (Promega, USA) with primers : 5′ -TTGCCGGTGGCCATCATGCACGTGGAGAAGGAC-3′ and 5′ -GT CCTTCTCCACGTGCATGATGGCCACCGGCAA-3′. The correction of all the constructs was confirmed by automated DNA sequencing.

**6. Western blotting**

Cells were lysed in Nonidet-P40 (NP-40) buffer (150 mM sodium chloride, 1.0% NP-40, 50 mM Tris, pH 8.0, 1×Roche protease and phosphatase inhibitor cocktail) with occasional vortex. The cell lysates were then centrifuged to remove debris at 15,000 rpm for 30 min at 4°C. The concentration of proteins in the lysates was determined by Bradford assay (Bio-Rad). Equal amounts of total protein for each sample was loaded and separated by 8%-12% SDS-PAGE and then transferred onto polyvinylidene difluoride (PVDF) membranes (Amersham Biosciences). Membranes were blocked with 5% Bull Serum Albumin (BSA) in TBST (20 mM Tris-HCl, pH 7.4, 150 mM NaCl, 0.1% Tween 20) for 1 h and incubated with specific antibodies. GAPDH was served as the loading control. Target proteins were detected with corresponding secondary antibodies (Santa Cruz Biotechnology, USA), visualized with a C600 western blot imaging system (Azure Biosystems, USA). Each immunoblot assay was carried out at least three times. The following antibodies were used in this study: anti-GAPDH (Santa Cruz, sc-47724), anti-PIM1 (CST, 3247), anti-Zika Envelope (GeneTex, GTX133314), anti-STAT2 (Santa Cruz, sc-514193), anti-pSTAT2 (Abcam, ab53132), anti-STAT1 (Abcam, ab47425), anti-pSTAT1 (Abcam, ab30645), anti-Zika NS5 (GeneTex, GTX133312), anti-Zika NS3 (GeneTex, GTX133309), anti-Zika NS1 (GeneTex, GTX133307), anti-Flag (ThermoFisher, MA1-91878).

**7. Viral RNA quantification**

The total cellular RNA was isolated for intracellular viral RNA quantification. To calculate the extracellular virions, the culture media of infected cells was firstly harvested and briefly centrifuged at 15,000 rpm for 30min at 4°C to remove cell debris. Viral particles were then precipitated with 10% polyethylene glycol 8000 containing 0.5 M NaCl at 4°C overnight. After centrifuging for 30 min at 16,000 g, viral particles were pelleted and treated with 100 μg/ml of RNase A (Sigma, USA). To isolate the intracellular virions, ZIKV infected cells were lysed with lysis buffer (1% Triton 100 and 1 x Roche protease inhibitor cocktail in PBS). The cell lysates were used to isolated viral particles as described above. Then the viral RNA was extracted from those infectious ZIKV viruses and quantified by RT-qPCR.

**8. Virus titration.**

Vero cells were seeded into 96-well plates for 24h before infection, then cells were infected with 100 μl of serial 10-fold diluted supernatant per well in quintuplicate. The 50% tissue culture-infected dose (TCID50) was calculated by the Reed-Muench method 5 days post infection.

**9.** **Immunofluorescence assay.**

RD cells were infected with ZIKV PRVABC59 at MOI 1. 24h post-infection, the monolayer of RD cells was fixed with 0.5% of paraformaldehyde (PFA) in PBS. Cells were blocked with 5% nonfat milk for 30 min at room temperature. Primary antibodies were incubated for 2h at room temperature after which the monolayer was washed three times with PBS. The samples were then reacted with rhodamine (tetramethyl rhodamine isothiocyanate [TRITC])-conjugated goat anti-rat IgG (Jackson ImmunoResearch Laboratories, Inc.) for 1h at room temperature. After washed with PBS, the samples were treated with DAPI for 5 min at room temperature and washed again with PBS three times. The monolayer was then washed three times with PBS. Cells were then visualized using an inverted fluorescent microscope (Nikon Eclipse Ti).

**10. RNA sequencing assay**

We treated HEK293T and A549 cells with PIM1 inhibitor CX-6258 at 4 μM for 24h for RNA sequencing assays. After 24h treatment, total RNA was harvested using Trizol reagent. This RNA sequencing and analysis were done by company (Novogene, Beijing). Briefly, Illumina TruSeq RNA Sample Prep Kit (Cat#FC-122-1001) was used with 1 ug of total RNA for the construction of sequencing libraries. RNA degradation and contamination were monitored on 1% agarose gels. RNA purity was checked using the NanoPhotometer® spectrophotometer (IMPLEN, CA, USA). RNA integrity was assessed using the RNA Nano 6000 Assay Kit of the Bioanalyzer 2100 system (Agilent Technologies, CA, USA). A total amount of 1 μg RNA per sample was used as input material for the RNA sample preparations. Sequencing libraries were generated using NEBNext® UltraTM RNA Library Prep Kit for Illumina® (NEB, USA) following manufacturer’s recommendations and index codes were added to attribute sequences to each sample. The library preparations were sequenced on an Illumina Novaseq platform and 150 bp paired-end reads were generated.

**11. Statistical analysis**

Results were expressed as mean ± standard deviation (SD). All statistical analyses were carried out with SPSS, version 16.0 software (SPSS Inc.). Two-tailed Student’s t test was applied for two group comparisons. A p value <0.05 was considered statistically significant.

**Supplementary Figure 1**

**Supplementary Figure 1. Stimulation of PIM1 expression by ZIKV**. (**a**) RNA-sequencing results of the KEGG pathway analysis of ZIKV-infected Vero cells. (**b**) RD and (**c**) A549 cells were infected with ZIKV at an MOI of 1 or 10 and incubated for 48 h. The protein levels of the PIM1 and ZIKV envelope proteins were determined by Western blotting (WB). GAPDH was used as the internal control.

**Supplementary Figure 2. Promotion of ZIKV replication by PIM1.** (**a**) PIM1 was silenced by two individual siRNA duplexes si-PIM1-1 and si-PIM1-2. The relative cellular PIM1 mRNA level was quantified by RT-qPCR at 48h after transfection in HEK293T cell. (**b**)The protein level of PIM1 was determined by West Blot assay (WB). GAPDH was used as the loading control. (**c**) PIM1 expression in A549 cells and (**d**) RD cells were first silenced, and then, the cells were infected with ZIKV at an MOI of 1 and incubated for 48 h. ZIKV envelope protein levels were determined by WB. (**e**) A549 cells and (**f**) RD cells with ectopic PIM1 expression for 48 h were infected with ZIKV at an MOI of 1 and incubated for another 48 h. The expression levels of ZIKV envelope protein and PIM1 were determined by WB. (**g, h**) RD cells were transfected with siRNAs for 24 h, and then infected with ZIKV at an MOI of 1 for 48 h. The levels of intracellular viral RNA (**g**) and extracellular virion RNA (**h**) were determined by RT-qPCR assay. (**i, j**) RD cells with ectopic PIM1 expression for 48 h were infected with ZIKV at an MOI of 1 and incubated for 48 h. The levels of intracellular viral RNA (i) and extracellular virion RNA (**j**) were determined by RT-qPCR assay. (**k**) PIM1 was silenced in the RD cells, then, the cells were infected with ZIKV at an MOI of 1 and incubated for 48 h. The viral protein envelope was observed by fluorescence microscopy. Red represents ZIKV envelope protein, and blue is DAPI. (**l**) siRNA targeting PIM1 3’-UTR at 40 nM was co-transfected with the PIM1 expression plasmid for 48h in RD cells, and then infected with ZIKV at MOI =1 48h. ZIKV envelope, NS1, NS3, NS5 protein expression level was determined. GAPDH was used as the internal control. Data are represented as the mean ± SD (n = 3). Student’s t test, * p<0.05, compared with the mock group; ** p<0.01, compared with the mock group.

**Supplementary Figure 3. Inhibition of ZIKV protein expression by PIM1 inhibitors.** The RD cells (**a, b, c**) and A549 cells (**d, e, f**) were treated with different PIM1 inhibitors CX-6258 (**a, d**), SGI-1776 (**b, e**) and AZD-1208 (**c, f**) at the indicated concentrations for 2 h, and then infected with ZIKV at an MOI of 1 and incubated for 48 h. DMSO (0.5%) was added to the control group. The envelope protein level was determined, and GAPDH was used as the internal loading control. (**g, h, i**) RD cells were treated with the indicated concentrations of CX-6258 (**g**), SGI-1776 (**h**) and AZD-1208 (**i**) for 2 h and then infected with ZIKV at an MOI of 1 for 48 h. The intracellular viral RNA levels were determined by RT-qPCR. GAPDH was used as internal control. Results are represented as mean ± SD from three independent experiments. * p<0.05, ** p<0.01.

**Supplementary Figure 4. Stimulation of the antiviral signaling pathway by inhibiting PIM1.** (**a**) HEK293T and A549 cells were firstly treated with 4 μM of CX-6258 for 24 h. The total RNA was extracted and applied for RNA-sequencing assay. The results are shown in a heat map. (**b**) A549 cells were treated for 24 h with the PIM1 inhibitor CX-6258 at the indicated concentrations. The mRNA levels of ISGs (APOBEC3H, APOBEC3C, PML, OASL and TRIM5) were determined by RT-qPCR. (**c**) PIM1were silenced by specific siRNA in A549 cells. The mRNA level of APOBEC3H, APOBEC3C, PML, OASL and TRIM5 were determined by qRT-PCR assay; GAPDH was used as internal control. (**d**) PIM1 was silenced in HEK293T cells by siRNAs. The relative phosphorylation of STAT1 and STAT2 was determined by WB. (**e**) HEK293T cells were transfected with PIM1 expressing plasmid for 48 h. The relative phosphorylation of STAT1 and STAT2 was determined by WB. (**f**) PIM1 was knocked down in RD cells for 48h. The p-STAT2 localization was observed by fluorescence microscopy. Red represents the p-STAT2 protein, and blue is DAPI. (**g**) PIM1 was silenced in HEK293T cells by RNA inference as explained above. The mRNA levels of IFN β , IFN α1 and IFN γ were determined by RT-qPCR. Results are represented as mean ± SD from three independent experiments. * p<0.05, ** p<0.01.
